# Fatigue Measurements in an Existing Highway Concrete Bridge

**DOI:** 10.3390/s22082868

**Published:** 2022-04-08

**Authors:** Harald Schuler, Martin Müller

**Affiliations:** School of Architecture, Civil Engineering and Geomatics, FHNW University of Applied Sciences and Arts Northwestern Switzerland, Hofackerstrasse 30, 4132 Muttenz, Switzerland; martin.mueller2@fhnw.ch

**Keywords:** bridge monitoring, maintenance, fatigue loads, truck passage

## Abstract

Knowing the actual fatigue effects helps to assess the structural safety of bridges more accurately. Especially when the actions change or increase, it is important to be able to determine this. This paper deals with the measurement and recording of actual fatigue loads at a critical point of an existing bridge. It shows how long-term effects can be separated from short-term effects and how amplitudes from passing trucks can be counted. The paper also highlights the challenges that arise when measuring actual fatigue loads.

## 1. Introduction

By monitoring critical points, the safety of infrastructure elements can be significantly increased. In long-term monitoring, changes in the measurand may indicate a potential for damage. Appropriate further investigations can then be initiated in good time. Damage events, like the one in Genoa [1,2,3], give reason to monitor bridges more intensively in the future [4,5,6]. High-frequency observations make it possible to determine the passage of individual heavy goods vehicles (HGVs). These pulse-like loads are crucial for the detection of fatigue loads, but their duration is only in the range of seconds. In order to be able to precisely record the amplitudes of the loads, they must be separated from the long-term influences. A sampling rate of a few tenths of a second is required for precise measurement, which can lead to large amounts of data being stored [7]. This publication describes the problem and an approach to solving it.

**Figure 1:**
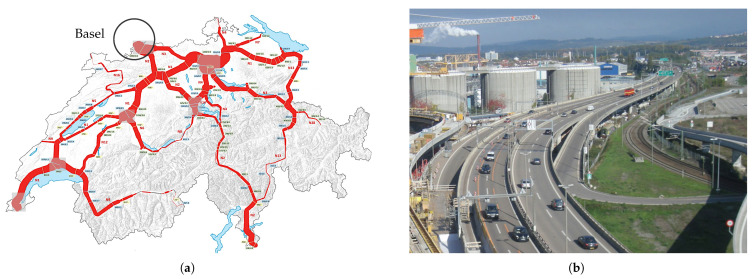
(**a**) The Swiss motorway network traffic in 2019, Basel location marked (https://www.astra.admin.ch/astra/de/home/themen/nationalstrassen/verkehrsfluss-stauaufkommen/verkehrsfluss-nationalstrassen.html (FEDRO), accessed on 6 April 2022); (**b**) Basel border bridge.

The A2 motorway connects Switzerland to Germany at the Basel border crossing. It is one of the busiest routes in Switzerland, being part of the main north–south connection to Italy. Figure 1a shows the relative traffic volumes on the motorway network in Switzerland. The border crossing to Germany represents an area of very high volume. In Basel, the traffic is routed over the border bridge, Figure 1b, with a total length of 1480 m. The bridge consists of 42 spans, each with a length of 35.4 m. After 38 years in operation, extensive renovation and reinforcement measures were necessary to repair damage and to enable the bridge to withstand increased loads in the future.

**Figure 2:**
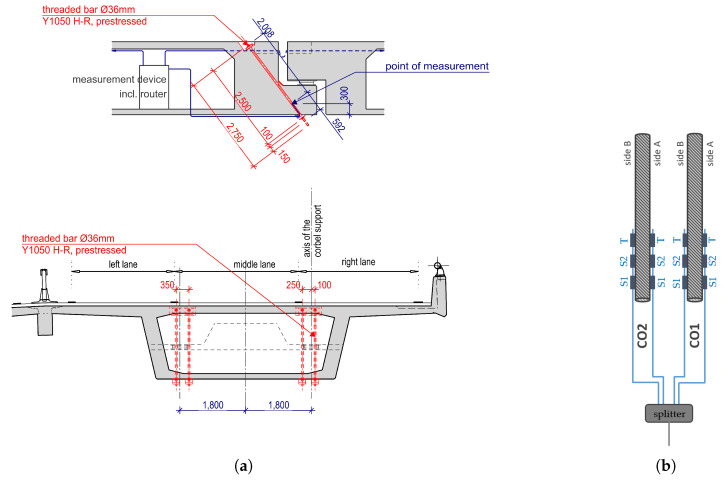
(**a**) Position of the pre-stressed threaded bar at the Gerber joint, cross-section of the bridge in one direction of travel; (**b**) strain (S1 and S2) and temperature (T) sensors on two parallel bars.

As part of these repairs, the Gerber joints were reinforced. These expansion joints serve to absorb the temperature deformations of the bridge. They are placed every 11th span, 390 m apart. The expansion joint itself consists of a corbel and a dapped beam end (see Figure 2a). The corbel was reinforced with two prestressed bars (⌀36 mm, Y1050) per web, each equipped with four strain and two temperature sensors, Figure 2b. Altogether, six bars were installed along the east side of the bridge. The optical sensors were glued onto rods with a diameter of 36 mm. Since the diameter of the drill hole, 50 mm is only slightly larger than the rod, there is just 8 mm of space on the side for protecting the optical sensors. Plastic angles were glued to both sides of the sensors to prevent damage during installation. Despite the tight conditions, more than two thirds of the sensors could be installed without damage and measure reasonable values. For more specific information about the fiber-optical sensors see [8]. The data is collected on site and then transmitted via mobile phone network to the server at the research institute, where it can be further processed and analyzed.

In [8], a new measurement setup for monitoring the corbels of the Basel border bridge was presented. As part of this project, it was determined that it was not possible to accurately calculate the stress peaks in the reinforcing rods due to passing trucks. Therefore, reference measurements were carried out and are described in detail in [8]. This article is about counting truck passages under regular operation to determine fatigue loads. For this purpose, long-term fluctuations had to be calculated out to obtain accurate strain peaks from truck passages. Then, the strain peaks, corresponding to specific truck loads, are assigned to different categories. The analysis covered one month and now forecasts for longer periods up to the remaining service life are possible. The calculation of load collectives from the monitoring data has not yet been done. For this topic, please refer to [9].

## 2. Reference Measurements: Correlation between Measured Strains and Truckloads

**Figure 3:**
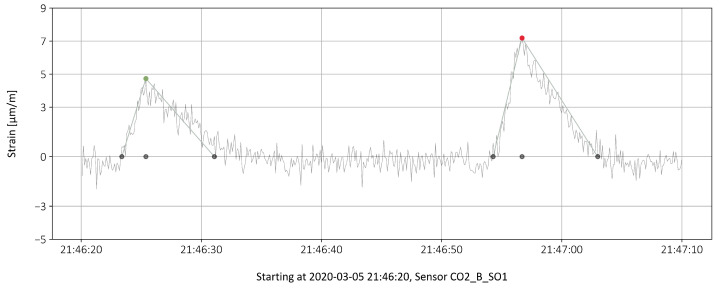
Reference measurement of the strain, a 24-tonne truck (left) followed by a 40-tonne truck, with points of interest marked.

In [8], reference measurements were made to obtain a correlation between the passing truck loads and the measured strains. Due to the special position of the sensors, it is possible to analyze the traffic on each of the two lanes of the bridge separately. A calculation of the strains from the loads is too complex and precise results cannot be calculated. A truss model was used to try to calculate the stress from the loads of the truck passages [7]. However, the results depend strongly on the compacted concrete around the prestressed rods, which can only be roughly estimated. A three-dimensional nonlinear finite element analysis could provide more insights, but the calculation is very complex when the concrete, slack reinforcement, and prestressed rods are to be modeled. Instead, reference measurements were made to establish a relationship between the load from passing trucks and the measured strains. Figure 3 shows the reference measurement at sensor C02_B_S01. The strain peak of the 24-tonne truck is clearly smaller than that of the 40-tonne truck.

Three categories are formed to distinguish between different load levels. An estimate of the corresponding truck weights is given in brackets:

– $3\;\mu m/m⩽\epsilon_{\mathrm{peak}}<5\;\mu m/m$(15t⩽truck weight<25t)
– $5\;\mu m/m⩽\epsilon_{\mathrm{peak}}<7\;\mu m/m$(25t⩽truck weight<35t)
– $7\;\mu m/m⩽\epsilon_{\mathrm{peak}}<9\;\mu m/m$(35t⩽truck weight<45t)

Compared to the reference measurement in Figure 3, the strains in the categories are set somewhat higher. This was adjusted because the driving speed in the reference measurement is much lower than in normal operation. In the reference measurement, the speed of the trucks was around 12 km/h. In regular operation it is between 50 km/h and 80 km/h. Therefore, a dynamic increase of about 10% is assumed for the normal case. Strains of less than 3 μm/m have not been taken into account, as in this range interference signals influence the measurement (background noise).

The peaks are characterized by 4 points, the start of the peak, the maximal value and the time position of that value and finally the end of the peak. The asymmetrical triangular shape of the peaks is explained in detail in [8].

## 3. Data Collection

The objective of this study is to record the actual fatigue loads on the bridge strengthening measure by counting the peak strains generated by heavy loads. The real fatigue loads usually differ considerably from the loads applied in the design, whereby no distinction is made between different amplitudes, but only the maximum amplitude is taken into account.

The collection of strain peaks is not straightforward, as temperature variations lead to much larger changes in strain than the loads generated by heavy traffic. However, the raw data can be transformed to reveal the almost imperceptible peaks caused by the passage of trucks. These strain peaks can then be assigned to one of the categories described in Section 3.2. All sensors on the bridge showed very similar patterns in the data. For reasons of uniformity, only the data from a single sensor will be considered further on.

### 3.1. Raw Strain Data and Preparation

**Figure 4:**
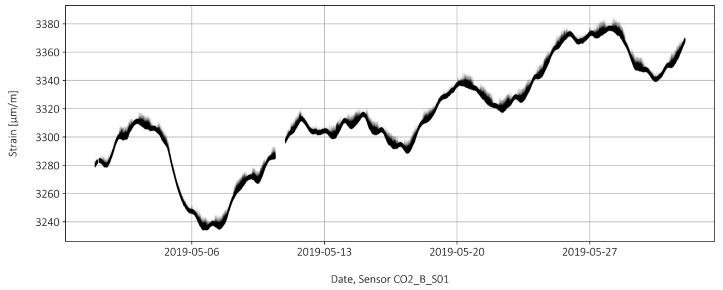
The raw strains measured at a single sensor over a period of one month.

The raw data of sensor C02_B_S01 is shown in Figure 4. For May 2019, a period of roughly 1.5 days (2019-05-10 09:47:04 to 2019-05-10 22:28:29) is missing due to an interruption of the power supply. Hence, the total recorded time was 29 days, 12 h, 41 min, and 25 s, which are about 29.5 days. A sampling rate of 10 Hz (one measure every 0.1 s) was chosen to capture the peaks accurately. As stated in [7], a sampling rate of 5 Hz (one measure every 0.2 s) is at least required to record an accurate profile for a truck passage. With an interval of 0.1 s, this results in a number of 25.5 million data points for each sensor for the month under consideration. In addition to the pre-tension, large fluctuations in the order of 140 μm/m can be observed, resulting only partially from temperature fluctuations (Section 3.2). In comparison, the amplitude due to the passage of a truck with a rolling weight of 40 tonnes is very small, approximately 8 μm/m. What looks like a thick line is actually a steady jitter around the line, which consists of 25 mill. points.

### 3.2. Temperature Measurement and Influence on Strain Data

**Figure 5:**
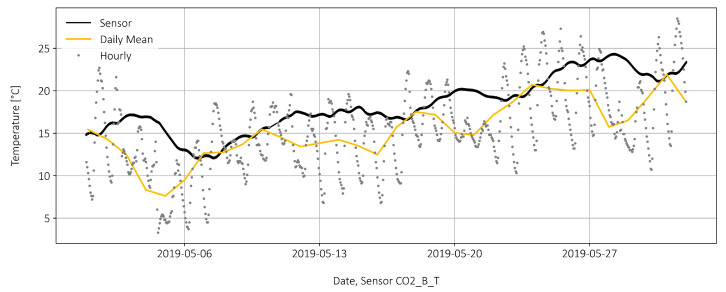
Temperature profile of the sensor in the bridge and temperatures from MeteoSwiss.

In addition to the four strain sensors, each reinforcement bar is also equipped with two temperature sensors. The temperature profile of the sensor C02_B_T is shown in Figure 5, where a steady increase is quite normal for this period of the year. The daily mean temperatures provided by the https://www.meteoswiss.admin.ch/home.html?tab=overview (Federal Office of Meteorology and Climatology MeteoSwiss) Basel are also displayed. The bridge temperature follows the air temperature, but with a time lag of two to three days. Hourly temperature fluctuations have only a minor effect on the temperature change inside the bridge. This is because the sensor itself is not exposed, but located in the lower third of the bridge superstructure and surrounded by at least half a meter of concrete in all directions, see also Figure 2a, where the location of the point of measurement is indicated in the side view of the Gerber joint.

**Figure 6:**
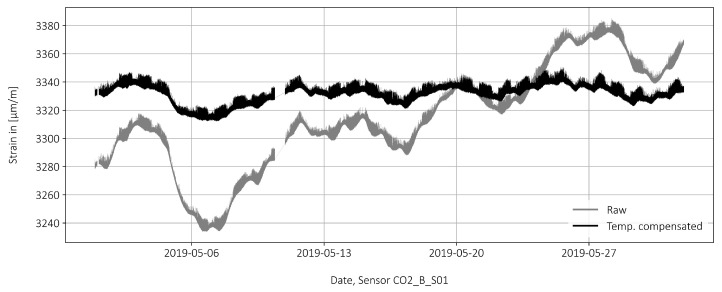
Raw strains from Figure 4 after temperature compensation.

The influence of temperature on the strain measurement is subsequently taken into account. The measured strains are compensated for the temperature strains. The strain sensors, for their part, are already temperature compensated. $T_{\mathrm{ref}}=20$ °C is used as the reference temperature at which no compensation takes place. If there is a deviation from the reference temperature, $\Delta T=T-T_{\mathrm{ref}}$, the strains are corrected by $\Delta \epsilon_{T}=-\Delta T\;\cdot \;\alpha_{T}$ with $\alpha_{T}=1\;\cdot \;10^{-5}/$°C. For example, an increase of +10 °C results in a strain correction of $\Delta \epsilon_{T}=-100\;\mu m/m$. Figure 6 shows the temperature compensated strains as well as the raw data without compensation. The long-term fluctuations without temperature compensation are in the range of 120 μm/m for the considered month of May 2019. After temperature compensation the fluctuations dropped to around 30 μm/m. However, these values are significantly larger than the strain peaks caused by the passage of a truck ( 8 μm/m for a 40-tonne truck). Therefore, a statistical method is used in Section 3.4 to highlight the strain peaks.

### 3.3. Normalizing the Strain Data

**Figure 7:**
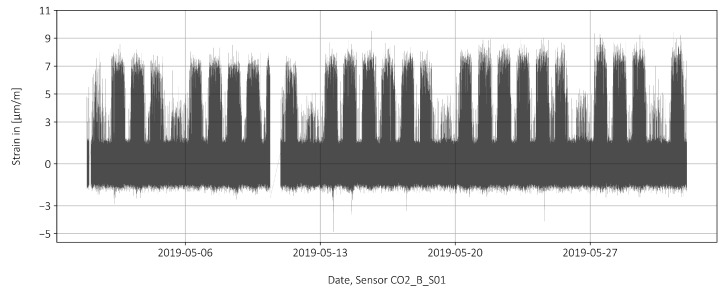
The normalized strain after applying a moving average smoothing.

Removing the long-term trend while retaining the short-term noise from the data can be accomplished by removing the long-term trend from the raw data, leaving only the short-term movement. The long-term trend was determined using a moving average over an interval of 295 data points, with 147 data points on either side of a moving central value. This 29-s time window proved to be long enough to remove the trend without squeezing off the peaks, matching the reference measurements described in Section 2. Figure 7 shows the data after trend removal with distinct large peaks on each weekday and Saturday, and only faint peaks on Sundays.

**Figure 8:**
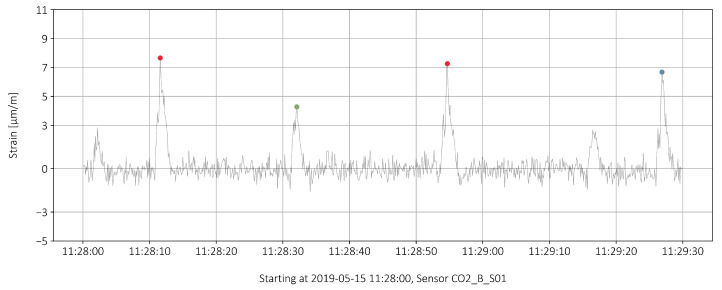
Categorized peaks over an interval of 90 s or 900 data points.

On closer inspection of the data in Figure 8, only the maximal value of each peak proved to be of interest, even more so for values larger than 3 μm/m as defined in Section 2.

### 3.4. Identifying and Counting the Strain Peaks

**Figure 9:**
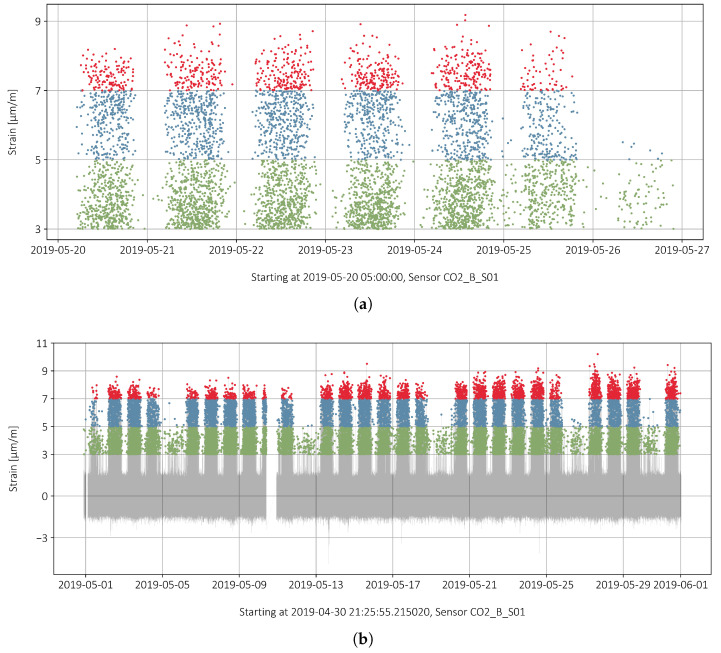
Strains and max. strains for different periods: (**a**) one week just peaks; and (**b**) one month with underlying normalized data.

A strain “peak” is defined as the maximal value measured over an interval where the strain is always positive and where the interval has a duration longer than 0.5 s. Figure 9a,b show the strain peaks for one week and the entire month. The colors correspond to the three categories defined in Section 2. There is a clear difference between weekdays and Sundays when a truck driving ban applies. The night driving ban from 10 p.m. to 5 a.m. is reflected in the gaps between the point columns. In Switzerland, a driving ban applies to all vehicles with a permissible total weight of more than 3.5 tonnes on Sundays and from 10 p.m. to 5 a.m.

The number of counted strain peaks in each category, as well as their mean value and standard deviation are given in Table 1.

**Figure 10:**
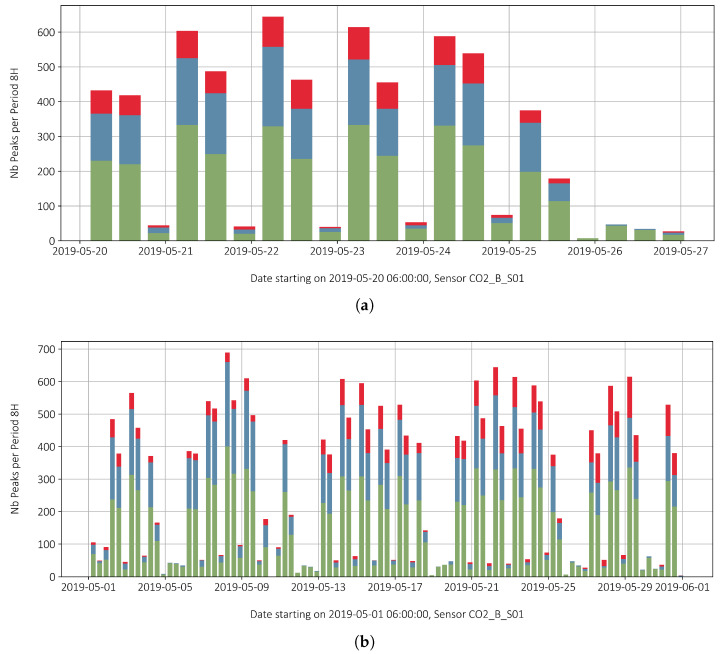
(**a**) Count of resampled peak strains for a duration of one week; and (**b**) resampled peaks for the entire month.

The high density of peaks makes it difficult to visualize the effective loads. Figure 10a,b show the peaks data stacked and resampled by time of day into 8-hour windows, as described hereafter:

– 6 a.m. to 2 p.m. (first column after date),
– 2 p.m. to 10 p.m. (second column after date),
– 10 p.m to 6 p.m. (third column after date).

Most of the traffic takes place in the first window. Between 2 p.m. and 10 p.m. the number is slightly lower, between 50 to 80 percent of the transit of the first period. Between 10 p.m. and 6 a.m., only very few passages are recorded, with most of them happening between 5 a.m. and 6 a.m., at a the time where the night driving ban does not apply. The first day in Figure 10a is a Monday. It should be noted that the proportions of the intensity of use (red, blue, green) remain roughly the same in the morning and evening and that highest counts occur on Tuesday, Wednesday and Thursday. Figure 10a thus gives a good overview of the times when the bridge is intensively loaded by heavy goods traffic.

Figure 10b gives an overview of the entire month of May, the first day being a Wednesday. Weeks without public holidays have similar amounts of stain peaks. Therefore, the week shown in Figure 10a can be considered representative of a typical working week.

**Figure 11:**
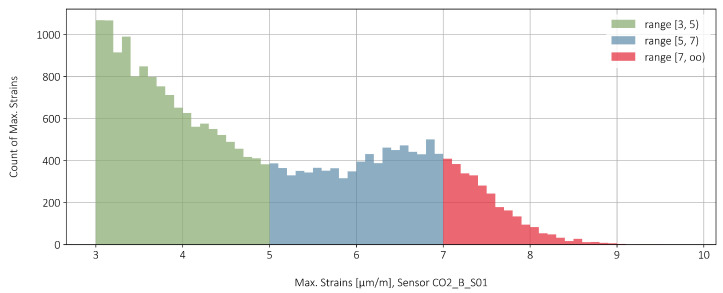
Histogram of the strain peaks for May 2019.

In Figure 11, the categories from Table 1 are shown as a histogram. A bin covers a strain range of 0.1 μm/m. In the category green and red the number of peak strains is decreasing with increasing amplitudes. Roughly speaking, one can say for the total measured data: the larger the strain amplitude, the less frequently it occurs in the measured range.

### 3.5. Comparison with Traffic Measurements

**Figure 12:**
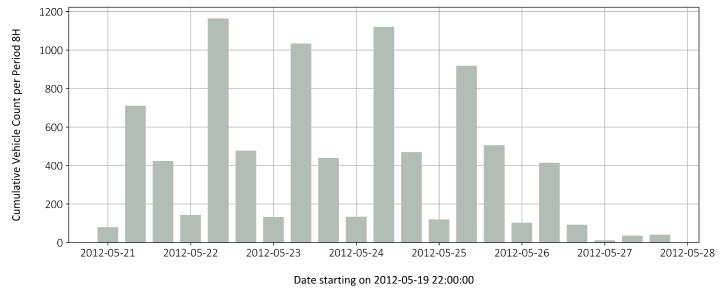
Heavy vehicle count during 4th week of May 2012, two lanes towards Germany.

To check the plausibility of the data, a comparison was made with traffic survey data. Unfortunately, the data for the Basel border bridge is only available up to 2012. Figure 12 shows the heavy goods traffic on the bridge for the fourth week of May 2012. This picture shows the traffic occurrence of both lanes, regardless of vehicle weight, because only the axle distance is measured. The traffic pattern is very similar and the numbers match rather well. A moderate increase in traffic will most likely continue.

In total, the average daily traffic, excluding Sundays, in 2012 towards Germany was 1207 heavy goods vehicles. On the lanes towards Germany, the share was significantly higher than in the direction to Basel. For 26 working days (including Saturdays) in May 2012, 33,130 heavy goods vehicles traveled to Germany over the bridge, but only 18,030 in the opposite direction. However, not all trucks were driving in the right lane and not all trucks were fully loaded. Only these trucks would have had a noticeable effect on the measurement. Comparing the 33,130 heavy goods vehicles from the traffic measurement (for both lanes) with the 24,380 from Table 1, the measurement appears to be within a reasonable range.

For a whole year, this results in a figure of 280 thousand trucks with a permissible total weight of more than 15 tonnes. The percentage distribution among the weight categories (20, 30 and 40 tonnes) can be seen in the Table 1.

## 4. Discussion

The monitoring of infrastructures at critical points can help to increase safety. Thereby for bridges, it is useful to distinguish between long-term and short-term monitoring. Long-term monitoring can detect gradual changes in a structure, that may indicate progressive damage. With short-term monitoring, vibrations or load pulses can be observed. Load pulses are mainly caused by heavy traffic and can lead to fatigue of structural elements. The number and amplitude play a decisive role. In the present study, these data are measured at a critical reinforcement point. Based on the evaluation, given for one month, a rough estimation for the entire service life could be made. As an example, the procedure is shown for one strain and temperature sensor. The same procedure can be used for all 24 strain and 12 temperature sensors on the 6 pre-stressed bars. The most important results of the study are listed below.

### 4.1. Raw Data and Influence of Temperature

A minimum sampling rate every 0.2 s is required to accurately capture the peak loads caused by passing trucks. In this study, a sampling rate of 0.1 s is used, resulting in 25.5 million data points for the month studied (May 2019 with an interruption of about 1.5 days). In [8], the shape of the impulsive load was analyzed for regular traffic and for the reference measurements. The pulses are approximately triangular with a steeper rise and flatter fall (see also Figure 3). The reason for this is that the measuring point is closer to the first support passed through than to the second. The duration of the pulse is less than 2 s at a truck speed of 60 km/h. In order to be able to record the maximum value (the amplitude) in a meaningful way, at least 10 points must be sampled in this time period. The strain measurement itself was temperature compensated. The following characteristics were observed in the measurement:

- The raw data of the strain measurement in May 2019 show global fluctuations of about 140 μm/m.
- The local fluctuations due to the passing of heavy goods vehicles are in a range up to about 10 μm/m. The amplitudes relevant for the evaluation are therefore many times smaller than the global fluctuations.
- The compensation of the temperature influence leads to a reduction of the fluctuations to about 30 μm/m. However, the residual fluctuations are still three times as large as the measured peak values caused by passing trucks. The reasons for the remaining fluctuations cannot be adequately explained. Therefore, the data for the measurement of the strain peaks are statistically processed.

### 4.2. Analysis of the Data and Counting Strain Peaks

To be able to precisely count the strain peaks due to the truck passing, the long-term effects were deducted. Using the statistical approach of the moving average, the fluctuation of the band can be subtracted so that only the short-time impulses remain. The following steps have been carried out:

- A window of 29 s (295 data points) was used to calculate the moving average. With this observation window, long-term fluctuations could be eliminated, and the short-term peaks remained.
- The counted load peaks were divided into three categories corresponding to rolling truck weights of about 20, 30 and 40 tonnes. With the measurement, detailed fatigue loads of the bridge could be determined. To verify the results, the data was compared with traffic measurements. The measured data were within a plausible range.

With the measurement system used and the proposed counting method for the pulse-like loads, detailed fatigue loads were recorded for one month. This data can be extracted to longer periods, such as service life without having to keep large amounts of data. Extrapolated from the counts, the number of load changes, assuming a 100-year lifetime, is 28 million for trucks weighing more than 15 tonnes. For trucks over 35 tonnes the number is 0.12·28 = 3.4 million, still higher than 2 million cycles at the fatigue threshold. Even for high peak loads, the number of load cycles is very high. This gives an impression of the actual fatigue load on the bridge. The fatigue check must be performed for each component individually. This is not the subject of this work, but it is about looking at the load situation. The research project has a total duration of 10 years until the year 2028, which also allows the observation of changes in loads on the bridge during this period. 

## Figures and Tables

**Table 1:** Count, mean value and deviation in each category of peak strain.

$\epsilon_{\mathrm{peak}}$ [μm/m]	Count	Percentage	Mean $\epsilon_{\mathrm{peak}}$	SD $\epsilon_{\mathrm{peak}}$
3–5	13,597	56%	3.82	0.56
5–7	7919	32%	6.05	0.58
7–*∞*	2864	12%	7.48	0.39
3–*∞*	24,380	100%		

## Data Availability

The data presented is not publicly available due to the funding agreement.
